# Quality of Life of Nursing Home Residents with Dementia: Validation of the German Version of the ICECAP-O

**DOI:** 10.1371/journal.pone.0092016

**Published:** 2014-03-14

**Authors:** Peter Makai, Franziska Beckebans, Job van Exel, Werner B. F. Brouwer

**Affiliations:** 1 Department of Geriatrics, Radboud University Medical Centre, Nijmegen, The Netherlands; 2 Siemens Health Insurance Fund, Munchen, Germany; 3 Institute of Health Policy and Management, Erasmus University Rotterdam, Rotterdam, The Netherlands; University of Glasgow, United Kingdom

## Abstract

**Objectives:**

To validate the ICECAP-O capability wellbeing measure’s German translation in older people with dementia living in a nursing home, and to investigate the influence of proxy characteristics on responses.

**Method:**

Cross-sectional study. For 95 residents living in a German nursing home, questionnaires were completed by nursing professionals serving as proxy respondents. We investigated the convergent validity of the ICECAP-O with other Quality of Life (Qol) measures, the EQ-5D extended with a cognitive dimension (EQ-5D+C), the Alzheimer’s Disease Related Quality of Life (ADRQL) measures, and the Barthel-index measure of Activities of Daily Living (ADL). Discriminant validity was investigated using bivariate and multivariate stepwise regression analysis, comparing ICECAP-O scores between subgroups varying in dementia severity, care dependency, ADL status and demographic characteristics.

**Results:**

Convergent validity between the ICECAP-O, EQ-5D+C, ADRQL and Barthel-Index scores was moderate to good (with correlations of 0.72, 0.69 and 0.53 respectively), but differed considerably between dimensions of the instruments. Discriminant validity was confirmed by finding differences in ICECAP-O scores between subgroups based on ADL scores (0.58 below 65 points on the Barthel-index and 0.80 above 65 points) and other characteristics. The ICECAP-O scores based on available tariffs were related to proxy characteristics gender (0.52 males versus 0.65 females) and work experience (0.61 below 2 years of experience versus 0.68 above 2 years).

**Discussion:**

The results of this study suggest that the ICECAP-O is a promising generic measure for general Qol and capability of people with dementia living in a nursing home. Validity tests generally yielded favorable results. Work experience and gender appeared to influence proxy response, which raises questions regarding appropriate proxies, especially since the ICECAP-O may be completed by proxies relatively often. Further research is necessary to validate the German version of the ICECAP-O, with specific attention to proxy completion for people with dementia.

## Introduction

Growing life expectancy leads to higher numbers of people with dementia due to increasing risk of incidence of dementia with age [1]. Currently, 5–7% of older people above 60 years suffer from dementia and this figure is expected to have doubled by 2030 [2]. Most people with dementia initially receive informal care at home, but with the progression of the disease, the amount of professional care typically increases. Frequently, in advanced stages of the disease, a sufficient amount and quality of professional care can only be provided in an institutional long-term care setting, making admissions inevitable for a growing number of people with dementia [3]. Faced with increasing demand, the long-term care sector in many countries may experience strong budgetary pressures, raising questions of optimal resource allocation and affordability of care.

Economic evaluation has traditionally assisted allocation decisions by integrally measuring health status and mortality using the QALY (Quality Adjusted Life Year) concept. In QALY calculations, values (often referred to as utility scores) are assigned to different health states, which allows the quantification of health gains comprising both length and quality of life gains from interventions [4]. These health states are commonly measured using Health-Related Quality of Life (HrQoL) instruments, which are used for computing the quality adjusted component of QALYs. This makes HrQol instruments an essential outcome measure for economic evaluation. Measurement of HrQoL is important for a chronic disease such as dementia, which impairs the quality of life of affected patients in addition to their length of life [5]. HrQoL is most commonly measured with the EQ-5D instrument [6]. Economic evaluation is increasingly used in the curative sector as a decision support tool for resource allocation, but may aid the allocation of resources in long-term care as well [6] [7] [8].

However, quality of life of individuals does not only depend on generically assessed HrQoL, as for instance measured by the EQ-5D, but also depends on other, non-health dimensions [4]. This is important in the context of economic evaluations when interventions do not (only) affect HrQoL but also these other factors of overall quality of life. For example, people with dementia living in nursing homes may have less contact with their family members, which may reduce their feelings of attachment and, consequently, general quality of life or well-being. Additionally, people suffering from advanced stages of dementia forget where they are, loose their sense of time and may no longer recognize their own family members [9] [10], which may lead to a decreased sense of control, and may inhibit their feeling of being valued. Therefore, to ensure a sense of accomplishment and independence for people with dementia, other activities matching their abilities and remaining resources are offered in nursing homes, for example through providing engaging activities [11] [12]. Such activities do not necessarily lead to an improvement in health, but will improve nursing home residents QoL more broadly by increasing their enjoyment of life, feeling of control and may contribute to a feeling of being valued. HrQoL instruments like the EQ-5D may not adequately reflect these elements of broader quality of life and therefore not be sufficient for a full economic evaluation of long-term care facilities [13].

Disease specific quality of life measures, such as the Dementia Quality of Life instrument (D-QOL) [14], the QuAlity of LIfe Measure for people with DEMentia (QALIDEM) [15], the Cornell-Brown Scale [16], the Qol-AD [17] and the Alzheimer Disease Related Quality of Life (ADRQL) [18] aim to capture such dementia-specific aspects of quality of life, with dimensions such as awareness of self and response to surroundings, feeling and belonging, and positive effect and negative affect [19]. Some dementia-specific outcome measures, such as the ADRQL have subscales and summary scores as well, translating a multidimensional HrQoL construct into a summary measure facilitating treatment comparisons [19]. However, by focusing on the effects of one particular disease, such measures may not capture the effect of other morbidities on HrQoL. This is of particular relevance to the nursing home population, where older people typically suffer from a range of co-morbidities [20], making it difficult to perform a complete assessment of the impact of specific interventions using disease-specific HrQoL instruments alone.

In order to be able to perform a complete economic evaluation the full benefit of the evaluated intervention or service should be measured. For this purpose, broader HrQoL measures, often named wellbeing measures, should be used to capture more facets of peoples’ lives than health status alone. A recently developed wellbeing instrument, the ICECAP-O (ICEpop CAPability measure for Older people), aims to incorporate such aspects beyond health [21] [22]. These broader wellbeing aspects are captured through the notion of capabilities, based on Amartya Sen’s capability approach [23]. Capabilities refer to the potential to achieve certain states and perform certain actions. Having the capability to live life the way one desires is obviously important, also to older people, and reduction of this capability limits their wellbeing [24] [23]. The ICECAP-O was originally developed to provide a set of general capability values –which thus differ from QALY values- for use in economic evaluations for people above 65 in the UK. Previous validation studies confirmed that the ICECAP-O evaluates a spectrum of outcomes beyond HrQoL [25] [21]. So far, the ICECAP-O has been used in the general population in the UK [25] and Australia [26], in a psycho-geriatric nursing home setting in the Netherlands [21], and among older adults with mobility impairments in Canada [27].

Measuring HrQoL and wellbeing in elderly suffering from dementia raises special challenges. At the stage of intermediate and advanced dementia the disease affects cognitive abilities and people lack the capacity of self-completing questionnaires (even in an interview setting) due to loss of memory, attention and language [18]. For all instruments in this study, we therefore used the proxy-report as suggested in the literature among people with moderate to severe levels of cognitive disorders [18] [28] [29] [30] [31] [32]. The choice of proxy may influence response, as professional and family proxies respond differently to HrQoL and wellbeing questionnaires in general [32] [33] and specifically for the ICECAP-O [21]. In case of psycho-geriatric residents, nursing professionals have been recommended as proxy to complete the ICECAP-O [21]. However, the influence of respondent characteristics beyond being a family member or a professional caregiver on the ICECAP-O remains unknown.

Measuring wellbeing is important in German long-term care as well. Around 1.3 million Germans suffer from dementia and this figure is expected to reach almost 2 million by 2040 [34]. In addition, institutionalization of people with dementia is quite common in the German context, and 40% of elderly with dementia are institutionalized [35] [10]. About 60% of nursing home residents in Germany suffer from dementia and require appropriate care [35]. Within the German long-term care system, three levels of care dependency are distinguished: low, medium and high care dependency, translating into a care requirement of daily assistance, 3 times assistance per day and 24 hours of care per day [36]. While levels of required care do not specify the location of care, mainly the second and third care dependency categories are represented in the institutional setting [36]. In addition, institutional care is seen mainly as a last resort when adequate care provision is no longer available or feasible at home due to social and familial circumstances or the severity of illness [36].

The aim of this study was to investigate the convergent validity and the discriminant validity - i.e., the ability to discriminate between subgroups, sometimes also termed clinical validity - of the ICECAP-O in a population of elderly with dementia living in a nursing home. Furthermore, we will explore whether proxy characteristics influence response.

## Methods

### Design, Setting, Study Population and Data Collection

We performed a cross-sectional study in two separate sites of a specialized nursing facility for dementia patients between May and August 2011 in North Rhine-Westphalia, Germany. The sample size consisted of 95 residents diagnosed with dementia, who were older than 55 and had been living in the nursing home for longer than two months. Nursing professionals (nurses, care assistants and nursing assistants) were selected as proxy respondents if they were primary caregivers of the dementia patient. Primary caregivers were defined as the persons who had the most experience with taking care of particular residents and were involved in their care at least four times a week. Nursing professionals were asked to complete the questionnaire in a manner as the client would have, if he/she would have been able to answer the questions. In total, 11 nursing professionals completed between 4 and 20 written questionnaires each. The Ethical Committee of the German Society for Nursing stated their formal approval was not required to conduct the study due to its non-invasive nature. Written informed consent was obtained from legal guardians for all 95 residents. To ensure privacy, the researchers did not see the name list of the residents at any time in the study.

### Measures

#### Dementia status

Dementia status was measured using the general practitioner’s diagnosis: type according to the ICD-10 (F00.-, F01.- or F02.-) [37] and severity according to the German guideline for dementia [38]. This classification is based on the Mini Mental Score Examination (MMSE), with mild dementia corresponding to MMSE scores between 20 and 26, moderate dementia corresponding to MMSE scores from 10 to 19 and severe dementia corresponding to MMSE scores below 10 [38]. Furthermore, care dependency (1 = low/2 = medium/3 = high care dependency) was measured using the care-level classification of the German National Association of Statutory Health Insurance Funds [36]. According to this classification, people in care level 1 need help once a day in some ADL activities, people in care level 2 need help three times a day, while people in care level 3 need continuous nursing care [36].

#### Wellbeing

The ICECAP-O measures capability wellbeing using five domains or attributes (attachment, security, role, enjoyment and control) and distinguishing four levels within each domain (levels generally range from all, lot, little, not any; exact wording of levels varies per dimension). The ICECAP-O thus distinguishes a total of 1,024 wellbeing states [4] [25]. The attributes were identified and formulated through extensive qualitative empirical research [24]. In order to obtain tariffs for the well-being states described with the ICECAP-O, the attributes were valued using best-worst scaling, a special type of discrete choice analysis [4]. The ICECAP-O tariffs have values between 0 (no capabilities) and 1 (full capabilities). In this study British tariffs were applied as German tariffs are lacking. For this first use of the ICECAP-O in Germany, the questionnaire was forward-backward translated from English into German by two independent translators.

#### Health-related quality of life

We used the revised 40-Item version of the Alzheimer Disease Related Quality of Life (ADRQL) instrument, which allows for the assessment of QoL for people with mild, intermediate or late-stage dementia using proxy response [18] [33] [14] [39] [40]. The dementia-specific, multi-dimensional ADRQL instrument can be completed by family members or patients professional caregivers [14] [5] [41] [42]. The ADRQL measures the dimensions Social Interaction, Awareness of Self, Enjoyment of Activities, Feelings and Mood, and Response to Surrounding [41]. The various dimensions range from 4 to 12 items on a dichotomous scale and each item is weighted in a range between 9.15 and 13.75, based on a judgment of importance by caregivers [43]. For each dimension a separate subscale can be calculated and summed up in one total score ranging from 0 (lowest quality of life) to 100 (highest quality of life) [44]. The instrument exhibits good psychometric properties having adequate validity, good internal-consistency reliability, very low missing data and good sensitivity to change [45] [46]. The authorized German edition of the ADRQL was used [42].

The EQ-5D as developed by the EuroQol group is a common instrument to measure generic HrQol [47]. The EQ-5D measures five dimensions (mobility, self-care, usual activities, pain/discomfort, anxiety/depression) on three levels (no problems, some problems, extreme problems) [47] [6], describing 243 health states. The EQ-5D health states can be converted to a utility score by applying the German EQ-5D index, based on TTO values [48] [49]. The EQ-5D utility scores range from 1 (perfect health) through 0 (dead) and has negative values accounting for health states worse than dead. For use in people with dementia, the EQ-5D was extended with a cognitive dimension, for which utility scores are unavailable [50] [51]. In this study the official German proxy version 2 of the EQ-5D was used [52] and a German translation of the question pertaining to the cognitive dimension was added.

#### Activities of daily living

The Barthel-Index is a well-established instrument that measures residents’ ability to perform activities of daily living (ADL) by proxy- or self-report. Decrease in ADL is one of the visible manifestations of dementia, and the subsequent loss of independence [53]. The ADL-score is mainly used in geriatric fields and is a strong predictor of QoL scores across several outcome measurements, including the ADRQL [33] [54]. The Barthel-Index includes items such as personal care and moving from wheelchair to bed and back, measured on two to four levels depending on the item. The available scores per question are 0 and 5 for two-level items, 0, 5, and 10 for three-level items and 0, 5, 10 and 15 for four level items, ranging from inability to independence. The total score thus ranges between 0 (completely dependent) and 100 (completely independent) [55] [56] with a cutoff score of 65 indicating need for ADL assistance [57]. In this study the validated German version was used [58].

#### Patient and proxy characteristics

Additionally, we collected data on patient’s age, sex, marital status, length of stay in the nursing home, and frequency of visits by family members. Finally, the questionnaire contained questions on age, role, work experience and length of time the nurse selected as proxy respondent knew the resident, since previous studies have shown that proxy characteristics may influence responses [32] [44].

### Hypotheses

To establish convergent validity we expected moderate to strong and positive correlations between the ICECAP-O, the EQ-5D and ADRQL scores because all of these instruments measure (partial) operationalizations of QoL (H1). Furthermore, we expected a moderate and positive correlation between the ICECAP-O dimension and tariff scores and the Barthel-index (H2).

For discriminant validity, we expected to find differences in ICECAP-O tariff scores between residents suffering from severe and mild/moderate dementia (based on the MMSE), between ADL dependent (Barthel score <65) and ADL independent (Barthel score ≥65) residents, between low, medium and high care dependency groups, and between older (75+ years) and younger (<75 years) residents (H3). A higher score on the ICECAP-O was expected for the better-off groups.

We expected that the proxy characteristics function (leading/non-leading), work experience (more or less than 2 years) and time knowing the resident (more or less than a year) would influence response on the ICECAP-O instrument (H4).

### Data Analysis

Descriptive statistics of resident and proxy characteristics were calculated. Correlations between the outcomes of the ICECAP-O and dimensions of the ADRQL, EQ-5D and the ADL were used to estimate convergent validity. Correlations above 0.5 were considered as strong, between 0.3 and 0.5 as moderate, and below 0.3 as weak [59]. Discriminant validity was analyzed using t-test and one-way-ANOVA to explore differences in means of the ICECAP-O between different demographic and dementia-related groups. Discriminant validity was also examined using two stepwise multivariate regressions, the first model controlling for patient variables (dementia severity, age, gender, time living in the nursing home, marital status, dementia type, ADL, frequency of visit, care level), and the second model for proxy characteristics as well (proxy gender, years of experience, function, time knowing the resident). For the stepwise analyses we used a cutoff of 0.1 for entering variables, using the forwards stepwise algorithm of STATA.

There was no missing data, so there was no need to correct for this in the study. For all analyses the level of significance was p<0.05. Data was analyzed using STATA 11.

## Results

### Descriptive Characteristics

Descriptive statistics of the 95 residents and the proxies are presented in Table 1. Average age of the residents was 77 years, with 54% being female and 55% of residents living in the nursing home for more than 2 years. 60% had Alzheimer’s dementia, and dementia severity could be categorized as severe in 60% of the cases. The majority of the residents (56%) had visitors less than once a week. As for the characteristics of the proxy respondents, the majority of the proxy respondents were female, and, on average, they had worked at the nursing home for 3.5 years.

**Table 1:** Demographic characteristics of residents and proxy’s (n = 95).

Variable		statistic
**Resident characteristics**		
Age		76.7 (8.5)
Sex (female)		56.8%
Type of dementia (Alzheimer’s)		60.0%
Dementia Severity	Mild	5.3%
	Moderate	34.7%
	Severe	60.0%
Length of stay in nursing home	0≤6 months	8.4%
	6≤12 months	13.7%
	12≤24 months	23.2%
	>24 months	54.7%
Marital Status	Unmarried	21.1%
	Married	23.2%
	Divorced	18,9.%
	Widowed	36.8%
Frequency of visits by family members	once a week or more	39.9%
	less than once a week	55.2%
	never	4.9%
Care Level	Level 1 (Low)	15.8%
	Level 2 (Medium)	33.7%
	Level 3 (High)	50.5%
**Proxy characteristics**		
Age		44.8 (11.5)
Sex (female)		87.0%
Working time (months)		43.4 (32.2)
Leading function		47.4%
Time knowing the resident (months)		19.2 (19.5)

**Figure 1:**
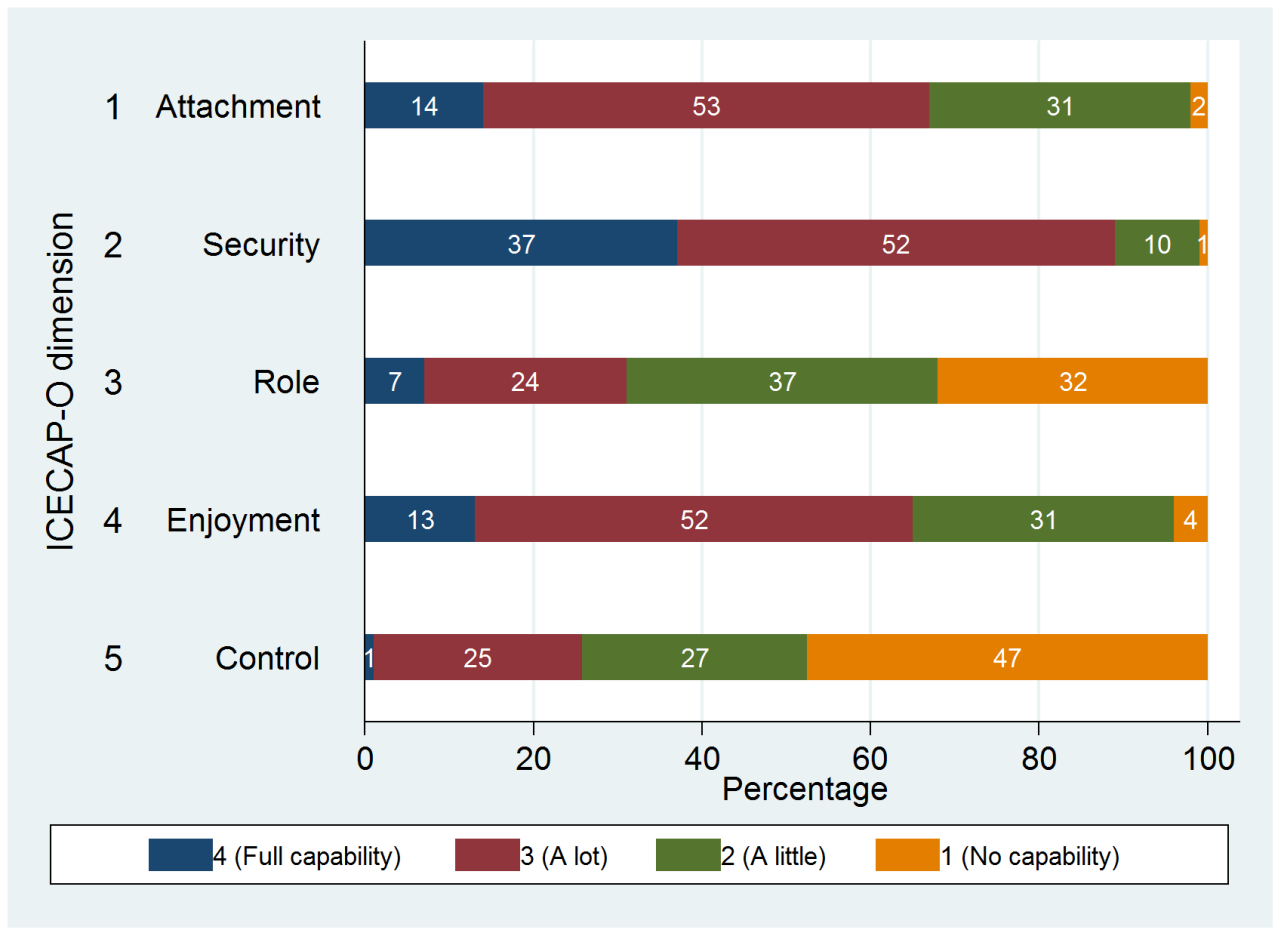
Response on the ICECAP-O.

Figure 1 illustrates the response to the ICECAP-O questions. On most dimensions, the majority of the residents had at least some deficits in terms of capabilities.

Table 2 describes the dimension and tariff scores of the measurement instruments used. The overall average scores for the instruments were as follows: average ICECAP-O score (based on the tariffs) was 0.63, EQ-5D score was 0.53, and the ADRQL score (based on tariffs) was 70.36.

**Table 2:** Description of measurement instruments (n = 95).

Instrument			Mean (SD)	Median
ICECAP-O	- scores based on tariff		0.63 (0.20)	
	- dimension scores	Attachment	2.79 (0.70)	3
		Security	3.24 (0.68)	3
		Role	2.07 (0.92)	2
		Enjoyment	2.73 (0.74)	3
		Control	1.78 (0.83)	2
Barthel-Index (ADL)	- score		41.18 (30.65)	
	- need for ADL assistance (73.7%with score <65)		27.21 (22.13)	
EQ-5D	- utilities		0.52 (0.34)	
	- dimension scores (+C)	Mobility	1.78 (0.87)	1
		Self-Care	2.52 (0.62)	3
		Usual activities	2.51 (0.56)	3
		Pain/Discomfort	1.35 (0.54)	1
		Anxiety/Depression	1.17 (0.43)	1
		Cognition (C)	2.69 (0.46)	3
ADRQL	- overall score		70.36 (15.69)	
	- dimension scores	Social Interaction (SI)	73.64 (26.63)	
		Awareness of Self (AS)	47.29 (28.19)	
		Feelings and Mood (FM)	83.83 (17.69)	
		Enjoyment of Activities (EA)	50.17 (28.69)	
		Response to Surroundings (RS)	90.56 (17.12)	

### Convergent Validity

Table 3 shows that the ICECAP-O scores were strongly correlated with EQ-5D scores, ADRQL scores and Barthel scores. Correlations between the ICECAP-O tariff scores and the different dimensions of the EQ-5D+C were generally strong and significant, except for the EQ5D+C dimensions “pain” and “anxiety”. Correlations between the ICECAP-O and the ADRQL proved to be similarly strong and significant, with the exception of the ADRQL dimensions “Feeling and Mood” (FM) and “Response to the Surroundings” (RS). The individual ICECAP-O dimensions Role and Control were strongly and significantly correlated with the EQ-5D+C dimensions mobility, self-care, usual activities and cognition. Role was also significantly and strongly correlated with AS (ADRQL). The Barthel index was significantly correlated with all ICECAP-O dimensions except for security, with correlations between the Barthel index and the role and control dimensions being particularly strong.

**Table 3:** Convergent validity (n = 95).

			ICECAP tariff	ICECAP dimension scores				
				Attachment	Security	Role	Enjoyment	Control
Barthel-Index (ADL)	- score		0.72**	0.25*	0.04	0.72**	0.40**	0.69**
EQ-5D	- utilities		0.69**	0.21*	−0.03	0.69**	0.35**	0.67**
	- dimension scores (+C)	Mobility	0.64**	0.17	0.00	0.59**	0.33**	0.60**
		Self-Care	0.61**	0.24*	−0.05	0.63**	0.27**	0.61**
		Usual activities	0.56**	0.24*	−0.09	0.54**	0.30**	0.58**
		Pain/Discomfort	0.23	−0.05	0.09	0.26**	0.13	0.25*
		Anxiety/Depression	0.16	0.27**	0.21*	0.09	0.26**	0.01
		Cognition (C)	0.48**	0.14	−0.07	0.52**	0.25*	0.54**
ADRQL	- overall		0.53**	0.48**	0.04	0.49**	0.56**	0.30**
	- dimension scores	Social Interaction (SI)	0.39**	0.43**	0.09	0.32**	0.46**	0.18
		Awareness of Self (AS)	0.56**	0.38**	−0.20	0.59**	0.43**	0.47**
		Feelings and Mood (FM)	0.13	0.14	0.15	0.10	0.27**	0.00
		Enjoyment of Activities (EA)	0.37**	0.28**	−0.09	0.34**	0.27**	0.23*
		Response to Surroundings (RS)	0.04	0.16	0.30**	0.02	0.15	−0.08

Note: *significance on the 5% level; **significance on the 1% level.

### Discriminant Validity

Table 4 shows the means of the ICECAP-O tariff scores in various subgroups defined by resident and proxy characteristics. The results of the t-tests showed significant differences in ICECAP scores between patients with different dementia severity (mild, moderate, severe), ADL scores (<65, ≥65) and ages (i.e., above or below 75). ANOVA results showed that the ICECAP-O tariff scores differentiated between residents classified into different care dependency levels. As expected, lower scores were observed for the more severe groups, and higher for the less severe groups. Additionally, the ICECAP-O tariff scores varied with two proxy characteristics: gender and work experience.

**Table 4:** ICECAP-O scores ability to discriminate between groups.

Variable	Level	Bivariate analysis			Stepwise multivariate analysis		
		Mean	SD	P-value	Beta	SD	P-value
**Resident characteristics**							
Age	75+	0.59	0.19	0.007			
	55–75	0.69	0.20				
Gender	Female	0.64	0.17	0.446			
	Male	0.63	0.23				
Dementia type	Alzheimer	0.61	0.19	0.103			
	Other	0.67	0.21				
Time in nursing home	<12 months	0.67	0.19	0.209			
	>12 months	0.62	0.23				
Marital Status	Married	0.65	0.22	0.400			
	Not married ^a^	0.63	0.19				
Visits	Once a week or more	0.63	0.19	0.361			
	Less than once a week	0.64	0.21				
Care level	Low	0.80	0.17	0.000			
	Medium	0.70	0.17				
	High	0.54	0.17				
Dementia severity	Mild/moderate	0.78	0.19	0.000			
	Severe	0.54	0.12				
ADL	Below 65	0.58	0.19	0.000	0.005	0.00	0.000
	Above 65	0.80	0.12				
**Proxy characteristic**							
Gender	Male	0.52	0.20	0.010	0.087	0.04	0.041
	Female	0.65	0.15				
Work experience	Less than two years	0.61	0.21	0.049	0.001	0.01	0.012
	More than two years	0.68	0.18				
Months knowing resident	Less than 12 months	0.63	0.21	0.474			
	Longer than 12 months	0.64	0.20				
Function	Leading	0.63	0.21	0.432			
	Non-leading	0.64	0.18				
R-square					0.55		

Note: ^a^ Unmarried, Divorced, Widowed.

Table 4 also shows the discriminant validity of the ICECAP-O tariff scores in a multivariate analysis. A relatively weak, but significant association was observed between the ICECAP-O tariff scores and ADL scores in both the model with only patient characteristics (analysis not shown) and in the model also including proxy characteristics. ADL coefficients, standard deviations and p-values were identical in both models. From the proxy characteristics, nursing professionals’ gender and work experience were associated with the ICECAP-O tariff scores.

## Discussion

### Main Results

In this study the ICECAP-O was used and validated for the first time in Germany, in a specialized nursing home for dementia patients. Our results indicate that the ICECAP-O has good convergent validity. Hypotheses were supported by the significant and strong correlations of the ICECAP-O tariff scores with HrQoL scores (both EQ-5D and ADRQL scores) (H1) and with ADL scores (H2). Moreover, as hypothesized (H3), the ICECAP-O significantly discriminated between subgroups based on dementia severity (mild, moderate and severe), ADL status (<65; ≥65), care level (low/middle/high) and age (residents younger and older than 75 years), thus supporting discriminant validity. In the stepwise multivariate model, the ICECAP-O discriminated between nursing home residents with different ADL status. The exploration of the relationship between the proxy responses on the ICECAP-O showed a significant influence of proxy characteristics on the ICECAP-O tariff scores (confirming H4).

### Methodological Limitations

Some limitations of this study deserve mentioning. First, residents all lived in two sites of the same nursing home facility and were not randomly selected. Therefore, they may have characteristics that differ from the typical population with dementia in German nursing homes. Hence, the results presented here are neither necessarily representative nor generalizable. However, the focus of the study was the validation of the properties of a wellbeing instrument in relation to various HrQoL instruments. For that purpose, the current sample seems adequate. Obviously, confirmation of these findings in other samples and settings remains important.

Second, at the time of this study, the ADRQL was the only dementia-specific instrument applicable to all stages of dementia available in German. Hence, this instrument was selected. In the meantime, however, a number of other measures have been validated in Germany, such as the Qol-Ad [60], the D-Qol [61], and the QALIDEM [62]. Further research to establish the convergent validity of the ICECAP-O with these instruments would be valuable.

Third, the sample size was relatively small. Hence, also in light of the promising results reported here, further research in larger samples is encouraged. Specific attention should also be paid in future research to the influence of proxy characteristics.

Fourth, nursing proxies completed varying numbers of questionnaires, which may have influenced our results. However, due to sample size considerations this could not be investigated in detail.

A fifth limitation is that only nursing proxies were used. Family members and spouses, who may assess residents’ QoL on the ICECAP-O differently than nursing professionals do [21], were not approached. It may be argued, that family members have a greater understanding of the individual and how they would perceive their own wellbeing since they will normally have known the patient for a much longer period of time as well as more profoundly. At the same time, family members are likely to compare the current state of the patients to their previous state(s), i.e. in relatively good health. This may induce them to focus more on the loss of health and wellbeing compared to before than on the current state for instance in comparison to other patients. Nursing professionals care for the residents on a day to day basis, at present have more contact with the residents than family members (frequently observing physical and mental conditions of patients, not only during visiting hours), and thus may be able to access the current QoL of elderly more accurately, also in comparison to other patients with dementia. Therefore, as suggested previously [21], in this care setting the nurse as proxy respondent seems to be the logical choice. Still, further research investigating the differences between family and nursing proxies is encouraged.

Finally, since German tariffs for the ICECAP-O were not available, British tariffs were used in this study. Although preference weights for capability dimensions may vary between countries, it is questionable whether using German tariffs (if available) would have led to different results regarding the validity of the ICECAP-O instrument. At the time of this study no ADRQL tariffs were available for Germany either, therefore we used the official American tariffs [43]. In order to investigate possible cultural effects on the valuation of the ADRQL, we performed a sensitivity analysis (results not shown) using weights from the German-speaking region of Switzerland, obtained in a pilot study [42]. Using these ADRQL weights in the sensitivity analysis did not yield different results.

### Convergent Validity and Discriminant Validity

The average ICECAP-O tariff score found in this study within a nursing home setting (0.63) was comparable to the score reported in a Dutch study performed in nursing homes (0.63) [21], and was substantially lower than the score for community-living elderly, where the average scores ranged between 0.81–0.84 [26], [27], [63]–[68]. These findings support the reliability of our results.

The strong correlation between the ICECAP-O and EQ-5D scores shows that generic HrQoL is captured to a wide extent by the ICECAP-O, which is consistent with other findings [25] [21] [26], [27], [63]–[68]. The results also confirmed the expected significant correlation between the ICECAP-O and the ADRQL scores, which shows that the ICECAP-O captures both generic HrQoL and dementia specific QoL. Additionally, the correlation between ADL and ICECAP-O scores reflected that a loss of independence in ADL was associated with a decline in wellbeing. Decreased ADL was also associated with lower scores on HrQoL instruments, confirming previous results that reduced ADL leads to a decrease in QoL [53]. Overall, these significant findings point in the direction of favorable convergent validity.

The ICECAP-O discriminated between patients based on the variables age, dementia severity, care dependency and ADL. This suggests that the ICECAP-O is sensitive to age differences and to indicators of health. In a multivariate setting, ICECAP-O scores were only significantly influenced by ADL, while dementia severity, care dependency and age were not significant. A possible explanation for this may be that ADL, dementia severity [69] and care dependency are related, while QoL is not necessarily determined by biological age per se. Dementia severity is one explanatory variable for the ADL status [69], which in turn determines care-dependency [36]. Another explanation for this finding may be a lack of power to detect all existing relationships between relevant variables. Indeed, in the bivariate analyses ICECAP-O scores varied with different dementia severity and ADL status, supporting discriminant validity of the German version of the ICECAP-O.

### Influences of Proxy Characteristics

That professional or family proxies provide different assessment of QoL has already been observed in other studies [32] [21] [28] [70]. However, specific proxy characteristics such as gender or work experience were not previously examined in relation to the ICECAP-O. Our results suggest that nursing professionals’ gender and work experience influence their response on the ICECAP-O.

Controlling for residents’ characteristics, proxy gender and work experience were related to the ICECAP-O tariff scores. In absence of a golden standard, it is difficult to judge which proxies provided the most accurate description of residents’ QoL. It may be hypothesized that in assessing QoL, nursing professionals benefit from more experience with caring for dementia patients. Male nursing professionals assessed residents QoL significantly higher than female nursing professionals did, controlling for ADL status of residents. This difference may either be due to the small number of questionnaires answered by male nursing professionals, or by a genuine gender difference in assessing residents’ QoL. The relationships between other proxy characteristics and proxy responses should be explored further in larger samples in future research. Such research should also address issues of inter-rater reliability between various proxies, such as professionals and family members of older people with dementia. Although a golden standard for the resident population included in this study is difficult to obtain, by comparing scores of proxies to those of patients obtained in early stages of dementia, one may perhaps shed more light on accuracy of QoL assessment of different groups of proxies.

### Conclusion

This study presented the first use of a German version of the ICECAP-O. The results indicate that the ICECAP-O appears to be a reliable wellbeing measurement instrument showing good convergent and discriminant validity for people with dementia. The influence of proxy characteristics like gender and work experience suggests potentially fruitful avenues for further research. In order to confirm the findings of this study, additional validation studies in larger samples and different settings are required.

Validating the ICECAP-O as a generic wellbeing instrument which has the capacity to capture broader outcomes might contribute to enabling economic evaluation of long-term care services and interventions, also in Germany. This seems to be especially relevant for informed decisions in the long-term care sector where an increase in healthcare spending is expected due to the growing number of elderly with dementia. In such a setting, appropriately measuring the potential benefits of care and comparing them to the costs is pivotal for optimal healthcare provision. By capturing the relevant outcomes in long-term care, the ICECAP-O seems to be a suitable wellbeing instrument for residents with dementia, though further validation work is encouraged.

## Supporting Information

Appendix S1 **The ICECAP-O instrument proxy version.**
(DOCX)

Appendix S2 **German version of the ICECAP-O.**
(DOCX)
