# Supplementary material for: Quality of Life of Nursing Home Residents with Dementia: Validation of the German Version of the ICECAP-O
Source: PLoS One. 2014 Mar 14;9(3):e92016. doi: 10.1371/journal.pone.0092016 (PMC3954837; doi:10.1371/journal.pone.0092016)
Supplement: Appendix S1 — The ICECAP-O instrument proxy version. (DOCX) [file pone.0092016.s001.docx]

# Supporting information 1: The ICECAP-O instrument proxy version

This appendix presents the ICECAP-O instrument proxy version used in this study. It was based on the original ICECAP-O version, as developed by Joanna Coast and Terry Flynn, and introduced and validated in [9]. The original version can be found at <http://www.icecap.bham.ac.uk/documents/icecapquest.doc>

General instructions for the proxy questionnaire: We would like to ask you to fill out the questions below for the client. Please try to answer the questions in manner as the client would if he/she would be able to answer the questions. With every question, please tick the answer that the client would give.

# ABOUT THE CLIENT’S QUALITY OF LIFE

By placing a tick (✓) in ONE box in EACH group below, please indicate which statement best describes the clients quality of life at the moment.

| 1. Love and Friendship |  |  |  |  | Tick **one**  **box**  **only in**  **each**  **section** |
| --- | --- | --- | --- | --- | --- |
| The client can have all of the love and friendship that he/she wants |  |  | **^4^** |  |  |
| The client can have a lot of the love and friendship that he/she want |  |  | **^3^** |  |  |
| The client can have a little of the love and friendship that he/she wants |  |  | **^2^** |  |  |
| The client cannot have any of the love and friendship that he/she wants |  |  | **^1^** |  |  |
|  |  |  |  |  |  |
|  |  |  |  |  |  |
| 2. Thinking about the future |  |  |  |  |  |
| The client can think about the future without any concern |  |  | **^4^** |  |  |
| The client can think about the future with only a little concern |  |  | **^3^** |  |  |
| The client can only think about the future with some concern |  |  | **^2^** |  |  |
| The client can only think about the future with a lot of concern |  |  | **^1^** |  |  |
|  |  |  |  |  |  |
|  |  |  |  |  |  |
| 3. Doing things that make you feel valued |  |  |  |  |  |
| The client is able to do all of the things that make him/her feel valued |  |  | **^4^** |  |  |
| The client is able to do many of the things that make him/her feel valued |  |  | **^3^** |  |  |
| The client is able to do a few of the things that make him/her feel valued |  |  | **^2^** |  |  |
| The client is unable to do any of the things that make him/her feel valued |  |  | **^1^** |  |  |
|  |  |  |  |  |  |
|  |  |  |  |  |  |
| 4. Enjoyment and pleasure |  |  |  |  |  |
| The client can have all of the enjoyment and pleasure that he/she wants |  |  | **^4^** |  |  |
| The client can have a lot of the enjoyment and pleasure that he/she wants |  |  | **^3^** |  |  |
| The client can have a little of the enjoyment and pleasure that he/she wants |  |  | **^2^** |  |  |
| The client cannot have any of the enjoyment and pleasure that he/she wants |  |  | **^1^** |  |  |
|  |  |  |  |  |  |
|  |  |  |  |  |  |
| 5. Independence |  |  |  |  |  |
| The client is able to be completely independent |  |  | **^4^** |  |  |
| The client is able to be independent in many things |  |  | **^3^** |  |  |
| The client is able to be independent in a few things |  |  | **^2^** |  |  |
| The client is unable to be at all independent |  |  | **^1^** |  |  |
|  |  |  |  |  |  |

© Original: Joanna Coast & Terry Flynn

**Tariffs and scoring algorithm in STATA**

The ICECAP-O has 5 attribute dimensions each having 4 levels, 44444 representing full capability and 11111 representing no capability. The tariffs for the ICECAP-O, based on the preferences of the 65+ population in the United Kingdom were presented by Coast et al. [8]. The tariffs basically provide preference weights for the different ICECAP-O states, which are normalised in such a way that 0 represents the worst situation described on the ICECAP-O (11111) and 1 represents the best situation described on the ICECAP-O. Lower scores thus represent fewer, preference based, capabilities.

The complete explanation as to how to calculate them is fully described on the ICECAP website: <http://www.icecap.bham.ac.uk/tariffs.shtml>.
