# Supplementary material for: Quality of Life of Nursing Home Residents with Dementia: Validation of the German Version of the ICECAP-O
Source: PLoS One. 2014 Mar 14;9(3):e92016. doi: 10.1371/journal.pone.0092016 (PMC3954837; doi:10.1371/journal.pone.0092016)
Supplement: Appendix S2 — German version of the ICECAP-O. (DOCX) [file pone.0092016.s002.docx]

# Supporting information 2: German version of the ICECAP-O

| **1. Liebe und Freundschaft** |  |  |  |
| --- | --- | --- | --- |
| Der Bewohner kann all die Liebe und Freundschaft haben, die er will |  |  | **^4^** |
| Der Bewohner kann viel von der Liebe und Freundschaft haben, die er will |  |  | **^3^** |
| Der Bewohner kann ein wenig von der Liebe und Freundschaft haben, die er will |  |  | **^2^** |
| Der Bewohner kann keinerlei von der Liebe und Freundschaft haben, die er will |  |  | **^1^** |
|  |  |  |  |
|  |  |  |  |
| **2. Gedanken über die Zukunft** |  |  |  |
| Der Bewohner kann über die Zukunft ohne Sorgen nachdenken |  |  | **^4^** |
| Der Bewohner kann mit wenig Sorgen über die Zukunft nachdenken |  |  | **^3^** |
| Der Bewohner kann über die Zukunft nur mit einigen Sorgen nachdenken |  |  | **^2^** |
| Der Bewohner kann über die Zukunft nur mit großen Sorgen nachdenken |  |  | **^1^** |
|  |  |  |  |
|  |  |  |  |
| **3. Dinge tun, durch die ich man sich geschätzt fühlt** |  |  |  |
| Der Bewohner ist in der Lage alle Dinge zu tun, durch die er sich geschätzt fühlt |  |  | **^4^** |
| Der Bewohner ist in der Lage viele Dinge zu tun, durch die er sich geschätzt fühlt |  |  | **^3^** |
| Der Bewohner ist in der Lage einige Dinge zu tun, durch die er sich geschätzt fühlt |  |  | **^2^** |
| Der Bewohner ist nicht in der Lage irgendwelche Dinge zu tun, durch die er sich geschätzt fühlt |  |  | **^1^** |
|  |  |  |  |
|  |  |  |  |
| **4. Freude und Vergnügen** |  |  |  |
| Der Bewohner kann all die Freude und das Vergnügen haben, die er will |  |  | **^4^** |
| Der Bewohner kann viele der Freuden und Vergnügen haben, die er will |  |  | **^3^** |
| Der Bewohner kann nur wenig der Freuden und Vergnügen haben, die er will |  |  | **^2^** |
| Der Bewohner kann keinerlei Freude und Vergnügen haben, die er will |  |  | **^1^** |
|  |  |  |  |
|  |  |  |  |
| **5. Unabhängigkeit** |  |  |  |
| Der Bewohner ist in der Lage, völlig unabhängig zu sein |  |  | **^4^** |
| Der Bewohner ist in der Lage, in vielen Dingen unabhängig zu sein |  |  | **^3^** |
| Der Bewohner ist in der Lage, in einigen Dingen unabhängig zu sein |  |  | **^2^** |
| Der Bewohner ist nicht in der Lage, unabhängig zu sein |  |  | **^1^** |
